# Peripheral Transcription of *NRG-ErbB* Pathway Genes Are Upregulated in Treatment-Resistant Schizophrenia

**DOI:** 10.3389/fpsyt.2017.00225

**Published:** 2017-11-06

**Authors:** Md Shaki Mostaid, Ting Ting Lee, Gursharan Chana, Suresh Sundram, Cynthia Shannon Weickert, Christos Pantelis, Ian Everall, Chad Bousman

**Affiliations:** ^1^Melbourne Neuropsychiatry Centre, Department of Psychiatry, The University of Melbourne and Melbourne Health, Parkville, VIC, Australia; ^2^The Cooperative Research Centre (CRC) for Mental Health, Melbourne, VIC, Australia; ^3^Centre for Neural Engineering, The University of Melbourne, Carlton, VIC, Australia; ^4^Florey Institute of Neuroscience and Mental Health, The University of Melbourne, Parkville, VIC, Australia; ^5^Department of Medicine, Royal Melbourne Hospital, Parkville, VIC, Australia; ^6^NorthWestern Mental Health, Melbourne, VIC, Australia; ^7^Department of Psychiatry, School of Clinical Sciences, Monash University and Monash Health, Clayton, VIC, Australia; ^8^Schizophrenia Research Institute, Sydney, NSW, Australia; ^9^Schizophrenia Research Laboratory, Neuroscience Research Australia, Sydney, NSW, Australia; ^10^Faculty of Medicine, School of Psychiatry, University of New South Wales, Sydney, NSW, Australia; ^11^Institute of Psychiatry, Psychology and Neuroscience, King’s College London, London, United Kingdom; ^12^Department of Medical Genetics, University of Calgary, Calgary, AB, Canada; ^13^Department of Psychiatry, University of Calgary, Calgary, AB, Canada; ^14^Department of Physiology and Pharmacology, University of Calgary, Calgary, AB, Canada

**Keywords:** treatment-resistant schizophrenia, *NRG–ErbB* pathway, gene expression, symptom severity, schizophrenia

## Abstract

Investigation of peripheral gene expression patterns of transcripts within the *NRG–ErbB* signaling pathway, other than neuregulin-1 (*NRG1*), among patients with schizophrenia and more specifically treatment-resistant schizophrenia (TRS) is limited. The present study built on our previous work demonstrating elevated levels of *NRG1* EGFα, EGFβ, and type I_(Ig2)_ containing transcripts in TRS by investigating 11 *NRG–ErbB* signaling pathway mRNA transcripts (*NRG2, ErbB1, ErbB2, ErbB3, ErbB4, PIK3CD, PIK3R3, AKT1, mTOR, P70S6K, eIF4EBP1*) in whole blood of TRS patients (*N* = 71) and healthy controls (*N* = 57). We also examined the effect of clozapine exposure on transcript levels using cultured peripheral blood mononuclear cells (PBMCs) from 15 healthy individuals. Five transcripts (*ErbB3, PIK3CD, AKT1, P70S6K, eIF4EBP1*) were significantly elevated in TRS patients compared to healthy controls but only expression of *P70S6K* (*P*_corrected_ = 0.018), a protein kinase linked to protein synthesis, cell growth, and cell proliferation, survived correction for multiple testing using the Benjamini–Hochberg method. Investigation of clinical factors revealed that *ErbB2, PIK3CD, PIK3R3, AKT1, mTOR*, and *P70S6K* expression were negatively correlated with duration of illness. However, no transcript was associated with chlorpromazine equivalent dose or clozapine plasma levels, the latter supported by our *in vitro* PBMC clozapine exposure experiment. Taken together with previously published *NRG1* results, our findings suggest an overall upregulation of transcripts within the *NRG–ErbB* signaling pathway among individuals with schizophrenia some of which attenuate over duration of illness. Follow-up studies are needed to determine if the observed peripheral upregulation of transcripts within the *NRG–ErbB* signaling pathway are specific to TRS or are a general blood-based marker of schizophrenia.

## Introduction

Intracellular signaling initiated by neuregulins (NRGs) and their cognate receptors (ErbBs) are vital for the assembly of neuronal circuitry (1, 2), including myelination of axonal processes (3, 4), neurotransmission (5), and synaptic plasticity (6–8). Abnormalities in *NRG–ErbB* signaling have been implicated in schizophrenia, with the majority of evidence linked to neuregulin-1 (*NRG1*) and *ErbB4* (5, 9–11).

**Figure 1:**
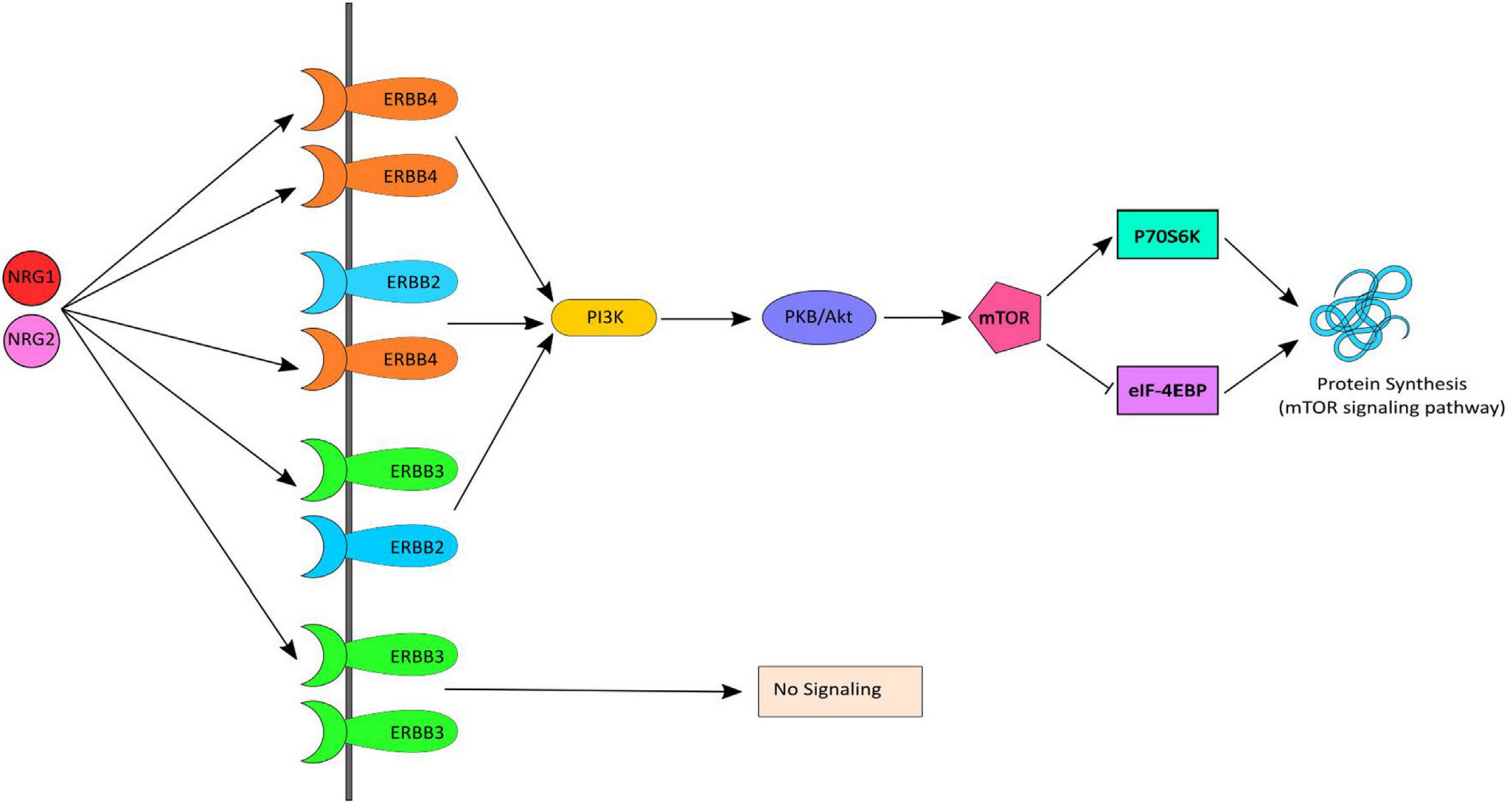
*NRG–ErbB* signaling pathway. Neuregulin-1 (*NRG1*) and *NRG2* bind to *ErbB3* and/or *ErbB4*, which in turn undergoes homo or heterodimerization and activates *PI3K*. *PI3K* then activates AKT and subsequently *mTOR* causing initiation of protein synthesis *via* the *mTOR* signaling pathway. mTOR phosphorylates and activates *P70S6K* which facilitates phosphorylation of small ribosomal protein 6 (*S6*) and eukaryotic translation initiation factor 4B (*eIF4B*) and leads to initiation of protein synthesis. Activated *mTOR* also causes phosphorylation and inactivation of *eIF4EBP1*, which release *eIF4E* and facilitates translation.

Neuregulin-1 and *ErbB4*, together, initiate signaling *via* the *PI3K-AKT* signaling pathway, which results in activation of *mTOR* and in turn stimulates protein synthesis (Figure 1). Several human postmortem brain studies have shown dysregulation of gene expression of *NRG1*, ErbB4 or down-stream targets among individuals with schizophrenia (12–17). Likewise, evidence of dysregulated gene expression of *NRG1* (18–20), *ErbB1*/*ErbB4* (21), and *PI3K*/AKT (22, 23) in peripheral tissues [i.e., whole blood, peripheral blood mononuclear cells (PBMCs), monocytes] in schizophrenia has also been shown in people with chronic schizophrenia. Treatment-resistant schizophrenia (TRS) patients represent a considerable subgroup who have significant increases in multiple *NRG1* splice variants in peripheral blood (24). Thus, we may expect the biological interactors (receptors) and mediators (kinase) of this pathway to also be changed. However, peripheral examination of gene expression within this pathway among individuals with TRS has yet to be completed. Moreover, the impact of medication, lifestyle (e.g., smoking, alcohol use), and/or symptom severity on *NRG1*-related mRNA expression is largely unknown.

The present investigation, therefore, quantiatively compared (i) whole blood mRNA levels of 11 *NRG–ErbB* signaling receptors and pathway genes (*NRG2, ErbB1, ErbB2, ErbB3, ErbB4, PIK3CD, PIK3R3, AKT1, mTOR, P70S6K, eIF4EBP1*) among individuals with TRS and healthy controls, (ii) associations between mRNA levels and symptom severity, age of onset, duration of illness, clozapine plasma level, and chlorpromazine equivalent dosage, and (iii) the effect of clozapine exposure on mRNA expression in PBMCs from healthy controls. We expected that there would be multiple molecular changes in TRS compared to controls that may contribute to the amplification of *NRG1* signaling in perhiperal blood in support of a widespread gain of function model of *NRG1* in the pathophysiology of schizophrenia.

## Materials and Methods

### Participants

#### Clinical Samples

Seventy-one participants aged 18–65 with schizophrenia who were treated with clozapine were recruited from inpatient and outpatient clinics in Melbourne, Australia. As these individuals failed to respond to two or more previous trials of antipsychotics, had poor functioning, and persistent symptoms, they were considered “treatment-resistant,” consistent with current criteria (25). In addition, 57 age-, sex-, and socioeconomic-matched unrelated healthy controls were recruited from the general community. Controls with a first-degree family history of psychiatric illness, prior or current use of antipsychotic medication, head injury, seizure, neurological disease, impaired thyroid function, and/or substance abuse/dependence were excluded. Detailed demographic characteristics of all participants are presented in Table 1.

**Table 1:** Demographic data and clinical characteristics of participants.

Characteristic	Schizophrenia (*n* = 71)	Controls (*n* = 57)	*P*-value
Age, mean (SD) years	40 (10)	40 (11)	0.702^a^
Gender, *n* (%) males	53 (75)	35 (61)	0.108^b^
RIN, mean (SD)	8.4 (0.9)	8.7 (0.3)	0.006^a*^
Ancestry, *n* (%) CEU	62 (90)	50 (88)	0.742^b^
Substance use in past 3 months, *n* (%)			
Tobacco (smoked)	33 (47)	12 (21)	0.003^b*^
Alcohol	59 (83)	55 (97)	0.016^b*^
Cannabis	11 (15)	7 (12)	0.385^b^
Amphetamine	4 (6)	2 (4)	0.439^b^
Cocaine	0 (0)	2 (4)	0.137^b^
Opiates	1 (1)	1 (2)	0.990^b^
Clozapine plasma level, mean (SD) μg/L	432 (234)	–	–
Chlorpromazine equivalent (excluding clozapine) dosage mean (SD) mg/day	142 (286)	–	–
Age of onset, mean (SD) years	22.5 (6)	–	–
Duration of illness, mean (SD) years	17 (8)	–	–
PANSS scores, mean (SD)			
Positive	10 (6)	–	–
Negative	15 (5)	–	–
Disorganized	8 (3)	–	–
Excitement	6 (2)	–	–
Depression	6 (3)	–	–
Total	62 (14)	–	–

*CEU, Northern and Western European ancestry; TRS, treatment-resistant schizophrenia; RIN, RNA integrity number; PANSS, Positive and Negative Syndrome Scale*.

*^a^Independent sample t-test*.

*^b^Chi-square (χ^2^) test*.

*P < 0.05.

Mini International Neuropsychiatric Interview (26) was administered to all participants to confirm the diagnosis of schizophrenia as well as to rule out the presence of psychiatric disorders in healthy controls. The Positive and Negative Syndrome Scale (PANSS) (27) was used to assess the clinical symptoms and the patients were scored in accordance with the consensus five-factor (i.e., positive, negative, disorganized/concrete, excited, depressed) PANSS model (28). Information on tobacco, alcohol, and illicit drug use in the past 3 months was collected using a substance use questionnaire. Whole blood samples were collected after overnight fasting and processed according to standardized blood collection and processing protocol (see supplementary methods for more details). Plasma levels of clozapine were measured and chlorpromazine equivalent dosage (excluding clozapine) were calculated for the 31% (*n* = 22) of participants with schizophrenia who were taking concomitant antipsychotic medication in accordance with published guidelines (29, 30). All the participants provided written informed consent and the study protocol was approved by the Melbourne Health Human Research Ethics Committee (MHREC ID 2012.069). The study complied with the Declaration of Helsinki and its subsequent revisions (31).

#### *In Vitro* Clozapine Exposure Samples

To assess the effect of clozapine exposure on gene expression of our candidate transcripts, fresh frozen PBMCs from 15 healthy individuals (8 males and 7 females) of European ancestry with a mean age of 35 (SD = 13.5; range 20–54 years) were purchased from STEMCELL™ Technologies, Inc. (Vancouver, BC, Canada). A sample size of 15 was sufficient to detect a large effect (Cohen’s *d* = 0.80) between exposed and unexposed conditions at α = 0.05 and power (1 − β) = 0.80. The percentage of current smokers among the donors was 33.3% (*n* = 5). All the donors were tested for HIV-1, HIV-2, hepatitis B and hepatitis C prior to blood collection.

Peripheral blood mononuclear cells isolated from whole blood were supplied as vials containing 100 million cells. PBMCs were rapid-thawed from liquid nitrogen and seeded in six-well plates in triplicates at a concentration of 2 million cells per well (1 × 10^6^ cells/mL) in RPMI-1640 medium (Sigma-Aldrich; St. Louis, MO, USA) supplemented with l-glutamine (0.3 g/L) and sodium bicarbonate (2 g/L), penicillin (100 U/mL), streptomycin (100 µg/mL), and 10% fetal bovine serum for 24 h. Cells were then exposed to clozapine (Sigma-Aldrich, St. Louis, MO, USA) for 24 h and 7 days, at a concentration of 1.2 µM (control cells were exposed to vehicle only, see supplementary methods for details) and incubated at 37°C in 5% CO_2_. Clozapine was initially dissolved in absolute ethanol and media was used for dilution. The final concentration of ethanol on each well was 1 in 8,000. The concentration of clozapine used was determined from the mean plasma concentration of clozapine found in the first 48 recruited clinical samples (1.2 µM or 384 ng/mL). Toxicity assays (CytoTox 96^®^ Non-Radioactive Cytotoxicity Assay; Promega Corporation, Madison, WI, USA) were performed at baseline, 24 h and 7-day time points after clozapine exposure to measure the production of lactate dehydrogenase within the media (see Figure S1 in Supplementary Material for more details).

### RNA Extraction, Complementary DNA (cDNA) Synthesis, and Quantitative Real-time PCR

PureLink RNA Mini Kit (ThermoFisher scientific, Waltham, MA, USA) was used to extract total RNA from both clinical and *in vitro* samples following standard manufacturer’s instructions. The RNA integrity number (RIN) range was 3.60–9.50 (mean = 8.59, SD = 0.79). Total RNA was reverse transcribed to complementary DNA (cDNA) using SuperScript^®^ IV First-Strand Synthesis System (Invitrogen, Foster city, CA, USA) using random hexamers. cDNA (10.25 ng) was used as a template for real-time PCR (RT-qPCR) using master-mix and gene specific validated Taqman assays from Applied Biosystems, Foster City, CA, USA. Inventoried assays (TaqMan^®^, Invitrogen, USA) were used for all the genes of interest as well as for four reference genes (beta-actin, ACTB; ubiquitin C, UBC; ABL proto-oncogene 1, ABL1; Succinate Dehydrogenase Complex Flavoprotein Subunit A, SDHA). See Table S1 in Supplementary Material for a list of each of the probes and primers.

Complementary DNA (10.25 ng) was subjected to quantitative real-time PCR in duplicate using FAM-MGB TaqMan^®^ gene expression probes (Invitrogen, Foster city, CA, USA) in 192 × 24 Dynamic Arrays IFC in Fluidigm^®^ BioMark™ HD system (South San Francisco, CA, USA) at the Monash Health Translation Precinct Medical Genomics Facility (Hudson Institute of Medical Research, Clayton, VIC, Australia). In addition, no reverse transcriptase controls and no template controls were included to rule out genomic DNA contamination and reagent contamination, respectively. Adhering to minimum information for publication of RT-qPCR (MIQE) guidelines (32), normalized relative quantities (NRQ), i.e., 2^−ΔC^*^t^* where Δ*C_t_* = [*C_t_*_(candidate gene)_ − *C_t_*_(geometric mean of reference genes)_] of each mRNA isoform was calculated using the geometric mean expression of two reference genes (UBC and ACTB) that did not differ between groups in the clinical cohort. ABL-1 and SDHA were not used as reference genes because their expression differed significantly by group in the clinical cohort (Figures S2–S4 in Supplementary Material). In the *in vitro* cohort only, ABL-1 was stable after 24 h clozapine exposure and ACTB was stable after 7 days clozapine exposure and were used for normalization and subsequent analysis at specific time points.

### Statistical Analysis

Two-sided tests were used for all statistical analyses. Shapiro–Wilk test and quantile–quantile (Q–Q) plots were used to assess normality of variable distributions. Student’s *t*-tests were used to test differences for continuous variables between schizophrenia patients and healthy controls, while chi-squared (χ^2^) tests were used for categorical variables. The Benjamini and Hochberg (B–H) step-up procedure (33) was used to adjust for multiple comparisons for all analyses. Effect sizes were calculated using the Hedges’ *g* method (34).

Prior to analysis, the NRQ values for all the mRNA transcripts were checked for normality using Q–Q plots (Figure S5 in Supplementary Material) and as required were log_10_ transformed for subsequent analysis. In addition, we assessed the following variables as potential confounders: age, sex, RIN, alcohol use, and smoking status. A variable was considered a confounder and included in our statistical models only when it was significantly different between groups (*P* < 0.05) and was significantly associated with gene expression. The log-transformed NRQ values were compared among groups using general or generalized linear models based on their distribution and adjusted for appropriate covariates. Outliers were identified using the Grubbs’ test for outliers and removed from further analysis.

Within the schizophrenia group, Pearson or Spearman correlations, depending on data distribution, were calculated between gene transcript levels and symptom severity, age of onset, illness duration, current chlorpromazine equivalent dose, and clozapine plasma levels. In addition, mRNA transcript levels between participants in positive symptom remission and non-remission were assessed using a *t*-test or Mann–Whitney *U* test. Positive symptom remission was defined as a PANSS score of ≤3 on delusions, hallucinations, grandiosity, and unusual thought content (28).

To assess differences in gene expression between clozapine exposed and unexposed PBMCs at both time points (24 h and 7 days), Wilcoxon matched paired *t*-test were used, adjusting for age, gender, and RIN.

## Results

### *NRG–ErbB* Signaling Pathway Transcripts Are Upregulated in TRS

**Figure 2:**
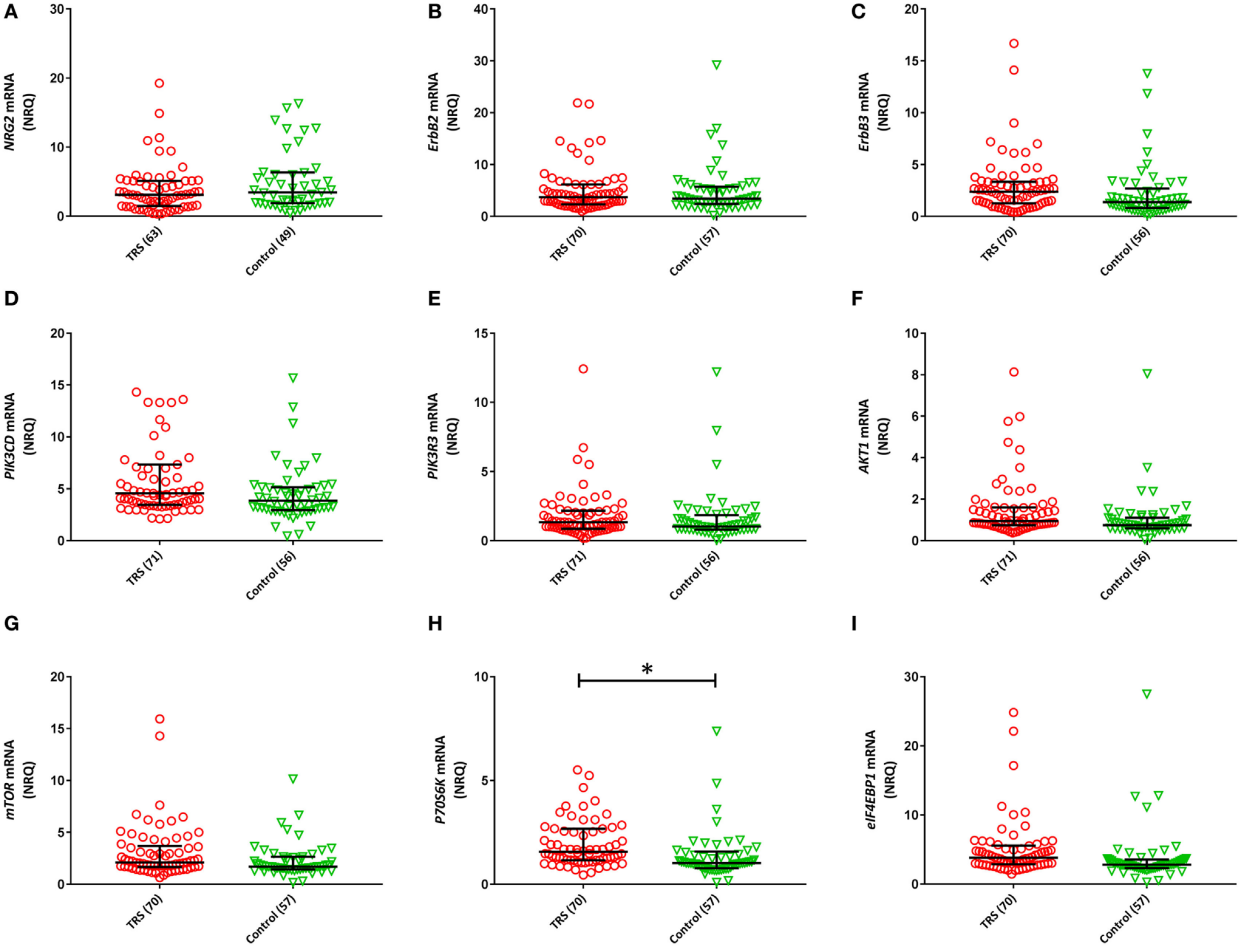
Normalized relative quantities (NRQ) of the gene transcripts: **(A)** *NRG2* [treatment-resistant schizophrenia (TRS): 3.11, interquartile range (IQR) = 1.5–5.12, controls: 3.44, IQR = 1.89–6.34; *F*_1, 111_ = 0.524, *P* = 0.113]; **(B)** *ErbB2* (TRS: 3.72, IQR = 2.39–6.19, controls: 3.44, IQR = 2.42–5.73; Wald χ^2^ = 0.029, *P* = 0.864); **(C)** *ErbB3* (TRS: 2.39, IQR = 1.26–3.35, controls: 1.38, IQR = 0.82–2.70; *F*_1, 126_ = 4.071, *P* = 0.083); **(D)** *PIK3CD* (TRS: 4.57, IRQ = 3.45–7.34, controls: 3.86, IQR = 2.94–5.16; Wald χ^2^ = 4.464, *P* = 0.079); **(E)** *PIK3R3* (TRS: 1.34, IQR = 0.86–2.17, controls: 1.02, IQR = 0.8–1.85; Wald χ^2^ = 0.104, *P* = 0.84); **(F)** *AKT1* (TRS: 0.94, IQR = 0.75–1.61, controls: 0.75, IQR = 0.59–1.11; Wald χ^2^ = 5.605, *P* = 0.054); **(G)** *mTOR* (TRS: 2.10, IQR = 1.66–3.69, controls: 1.44, IQR = 1.44–2.65; Wald χ^2^ = 4.746, *P* = 0.20); **(H)** *P70S6K* (TRS: 1.57, IQR = 1.16–2.68, controls: 1.02, IQR = 0.77–1.58; Wald χ^2^ = 13.90, *P* = 0.018); **(I)** *eIF4EBP1* (TRS: 3.81, IQR = 2.88–5.58, controls: 2.80, IQR = 2.30–3.55; Wald χ^2^ = 8.71, *P* = 0.054). Error bars represent median with interquartile range. Benjamini–Hochberg adjusted *P*-values are shown (*P < 0.05).

Two (*ErbB1, ErbB4*) of the 11 *NRG–ErbB* pathway mRNA transcripts interrogated, were not detectable in more than 80% of the full cohort and so were removed from further analysis. The rates of non-detects were not significantly different between groups (*ErbB1*: case 95%, control: 97%; *ErbB4*: case 81%, control 85%). Analysis on the remaining nine transcripts showed significantly elevated levels of five transcripts: *ErbB3* (*P* = 0.046), *PIK3CD* (*P*_raw_ = 0.035), *AKT1* (*P*_raw_ = 0.018), *P70S6K* (*P*_raw_ = 0.002), and *eIF4EBP1* (*P*_raw_ = 0.013) in TRS patients compared to healthy controls after adjustment for covariates. However, only *P70S6K* (*P*_B–H_ = 0.018) remained significant after correction for multiple comparisons (Figure 2). Importantly, transcript levels were not correlated with clozapine plasma levels or chlorpromazine equivalent antipsychotic exposure (excluding clozapine) (Table S2 in Supplementary Material). The lack of relationship between mRNA levels and clozapine levels were further corroborated by our *in vitro* analysis that showed no difference in mRNA levels of detectable transcripts (*n* = 9) in clozapine exposed compared to unexposed PBMCs, except *mTOR* mRNA which showed decreased expression levels in clozapine exposed cells at both 24 h (*P* = 0.001) and 7-day (*P* = 0.05) time points (Figures S6 and S7 in Supplementary Material).

### *NRG–ErbB* Signaling Pathway Transcripts Are Associated with Duration of Illness but Not Age of Onset or Symptom Severity

**Figure 3:**
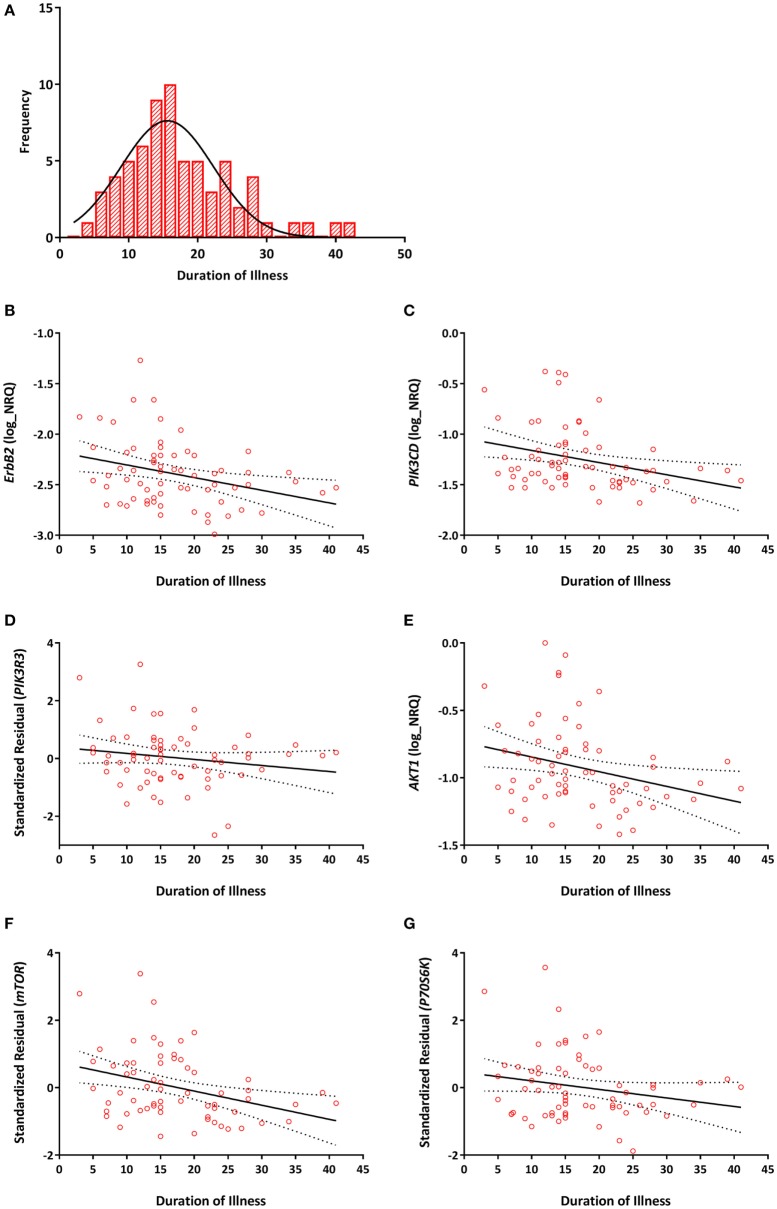
**(A)** Distribution of duration of illness in years (mean = 17, SD = 8). Correlations between duration of illness and **(B)** *ErbB2* (*r* = −0.293, *P*_B–H_ = 0.031); **(C)** *PIK3CD* (*r* = −0.303, *P*_B–H_ = 0.031); **(D)** *PIK3R3* (*r* = −0.275, *P*_B–H_ = 0.038); **(E)** *AKT1* (*r* = −0.290, *P*_B–H_ = 0.031); **(F)** *mTOR* (*r* = −0.339, *P*_B–H_ = 0.023); **(G)** *P70S6K* (*r* = −0.347, *P*_B–H_ = 0.023) mRNA expression. Expression of *PIK3R3, mTOR*, and *P70S6K* are represented as the standardized residual from a linear regression model after adjusting for potential confounds [i.e., age for *PIK3R3*, RNA integrity number (RIN) and smoking for *mTOR*, age, RIN and smoking for *P70S6K*]. Solid lines represent the line of best fit and dotted lines represents 95% confidence intervals for the line of best fit.

Among individuals with TRS, significant negative correlations between duration of illness and *ErbB2* (*r* = −0.293, *P*_raw_ = 0.016, *P*_B-H_ = 0.031), *PIK3CD* (*r* = −0.303, *P*_raw_ = 0.013, *P*_B-H_ = 0.031), *PIK3R3* (*r* = −0.275, *P*_raw_ = 0.025, *P*_B-H_ = 0.038), *AKT1* (*r* = −0.290, *P*_raw_ = 0.017, *P*_B-H_ = 0.031), *mTOR* (*r* = −0.339, *P*_raw_ = 0.005, *P*_B-H_ = 0.023), and *P70S6K* (*r* = −0.347, *P*_raw_ = 0.005, *P*_B-H_ = 0.023) expression were detected (Figure 3). None of the reference genes were significantly correlated with duration of illness, *UBC* (*r* = −0.139, *P*_raw_ = 0.263), *ACTB* (*r* = 0.232, *P*_raw_ = 0.59). No significant correlations were observed between any of the transcripts and age of onset (Table S2 in Supplementary Material).

A significant positive correlation between *ErbB2* expression and PANSS excitement score (*r* = 0.289, *P*_raw_ = 0.014, *P*_B–H_ = 0.667) was observed but did not survive correction for multiple comparisons (Table S3 in Supplementary Material). An exploratory examination of TRS patients in positive symptom remission versus non-remission revealed no statistically significant differences in levels of any of the gene mRNA transcripts after correction for multiple comparisons (Table S4 in Supplementary Material).

## Discussion

Our findings suggest transcription in the *NRG–ErbB* signaling pathway is upregulated in the whole blood of individuals with TRS and is negatively correlated with duration of illness. Among the nine detectable *NRG–ErbB* pathway transcripts we examined, five (*ErbB3, PIK3CD, AKT1, P70S6K*, and *eIF4EBP1*) were elevated and, of these, *P70S6K* survived correction for multiple comparisons. Importantly, we could not attribute this upregulation of peripheral transcription in the *NRG–ErbB* pathway to age, sex, or medication. In fact, results from our *in vitro* clozapine exposure experiment suggested clozapine might reduce rather than increase transcription of genes within the *NRG–ErbB* signaling pathway, particularly *mTOR* expression. Overall, our findings support our hypothesis that there is a generalized increase in *NRG1* signaling in people with TRS.

Previous findings by us and others support the notion of increased transcription of genes within the *NRG–ErbB* signaling pathway in schizophrenia. We recently showed in the same cohort used in the current study, an increased expression of three *NRG1* transcripts [i.e., *NRG1*-EGFα, *NRG1*-EGFβ, and *NRG1*-typeI_(Ig2)_] in TRS compared to controls (24). In addition, several studies by others have reported increased expression of specific isoforms of *NRG1* (18) and mRNA of down-stream signaling molecules, including *PIK3CD, PIK3CB* (16, 22), and *AKT1* (22, 23) in schizophrenia patients. Furthermore, other down-stream signaling molecules, such as *mTOR, P70S6K*, and *eIF4B*, have been shown to be increased in major depressive disorder (35). However, as we are not aware of any human studies that have interrogated *P70S6K*, in schizophrenia, we are the first to report increased mRNA of *P70S6K* in TRS.

*P70S6K* encodes for a vital kinase in the mTOR signaling pathway (36–38) that when phosphorylated by mTOR results in phosphorylation and activation of translation elongation factors *eIF4B* and *eEF2K*, thereby promoting protein translation (39, 40). Our findings suggest upregulation of *P70S6K*, in part, may result from an increase in transcription of several genes upstream of *P70S6K* within the *NRG–ErbB* signaling pathway. However, other genes (i.e., *BDNF, DISC1*) as well as neurotransmitters (i.e., glutamate, serotonin) and hormones (e.g., insulin) have also been shown to activate the *PI3K–AKT–mTOR* signaling pathway (41–43) and as such may contribute or confound the increase in *P70S6K* expression we have observed. However, most studies find decreased BDNF levels in the blood of people with schizophrenia (44) and suggest some degree of insulin resistance in clozapine-treated patients (45). Future investigations should attempt to account for these other signaling factors and the potential confounders of metabolic changes in people with schizophrenia being treated with clozapine, as doing so will further elucidate the suitability of *P70S6K* as a peripheral biomarker of over-activity in the *NRG1* pathway in schizophrenia.

We also detected trend-level increases in three transcripts (*ErbB3, PIK3CD*, and *AKT1)* upstream of *mTOR*, within the *NRG–ErbB* signaling pathway among those with TRS. These increases in whole blood expression are, in part, supported by previous studies that have shown an increased *AKT1* mRNA expression in PBMCs from individuals with early-onset (23) and treatment-naïve schizophrenia (46), suggesting peripheral upregulation of *NRG–ErbB* pathway transcripts may not be specific to the stage of illness and may occur during the first phases of schizophrenia and continue during the chronic phases. However, six of the mRNA transcripts (*ErbB2, PIK3CD, PIK3R3, AKT1, mTOR*, and *P70S6K*) we examined were negatively correlated with duration of illness, suggesting that as the illness progresses the upregulation of transcription within the *NRG–ErbB* signaling pathway might become less apparent. However, it is not clear whether this correlation represents a potential disease process and/or a compensatory response in an effort to maintain signaling homeostasis. Studies examining patterns of *NRG–ErbB* signaling pathway transcripts over the course of the illness are required to confirm this notion and determine the underlying mechanism.

We did not find differences in the peripheral expression of *NRG2* between TRS patients and controls. To our knowledge, we are the first to examine *NRG2* mRNA in the blood in schizophrenia or other psychiatric disorder. However, a recent study showed that ablation of *NRG2* in the adult mouse brain mimicked dopaminergic imbalance seen in schizophrenia (i.e., high subcortical dopamine, low cortical dopamine) and resulted in severe behavioral phenotypes relevant to psychiatric disorders (47). Thus, *NRG2* may play a role in the pathophysiology of schizophrenia but based on our results seems less likely to serve as a peripheral marker of neurobiological changes found in schizophrenia. Likewise, *ErbB2* mRNA expression seems an unlikely peripheral marker of schizophrenia based on our null findings as well as findings from others that reported no difference in *ErbB2* mRNA expression in monocytes of first-episode, drug-naive patients with schizophrenia compared to healthy controls (48). However, this same study suggested that there may be an exaggerated *NRG1* stimulated cytokine response from PBMC in people with schizophrenia compared to controls (48), suggesting a link between overactive *NRG1* signaling and inflammation.

Our study has notable limitations. First, we were unable to compare affected individuals with and without TRS and as such the specificity of our results to TRS patients remains to be confirmed. Second, we analyzed cross-sectional data, which makes it complicated to predict how gene expression patterns might change with disease progression and their possible relation to clinical symptoms. Third, we measured gene expression in whole blood, as this tissue is clinically accessible and commonly used in biomarker research. However, it is unclear how our findings will relate to other peripheral (PBMCs or lymphocytes) or central tissues (e.g., brain) despite some suggestion for their relevance in schizophrenia (49). Fourth, we did not investigate all transcripts within the *NRG–ErbB* pathway (i.e., *PIK3CA-B, PIK3R1-2, eIF4B, eEF2*, and *eIF4E*). We instead, chose transcripts based on evidence from the current literature in schizophrenia. Furthermore, we only interrogated mRNA levels of our candidate genes within the *NRG–ErbB* pathway and as such cannot rule out the potential that genetic, protein, and/or epigenetic markers in this pathway may differ in those with schizophrenia. Fifth, our sample size was relatively small and as such requires independent validation. Finally, our *in vitro* clozapine exposure experiments examined a single clozapine concentration (1.2 µM) that was guided by pilot data from our study population. While this concentration of clozapine does reflect steady state plasma concentrations (50–52), future work with PBMCs should examine multiple concentrations that reflect the range of clozapine blood levels observed in the clinic together with interrogating a greater number of candidates at both genetic, gene expression and protein levels.

In summary, our results provide the first peripheral gene expression profile of the major *NRG–ErbB* pathway genes among individuals with TRS. We detected an overall upregulation of *NRG–ErbB* pathway transcripts among those with TRS, most robustly for *P70S6K*. We further showed that most of the transcripts we examined were negatively correlated with duration of illness, suggesting the upregulation of *NRG–ErbB* pathway transcripts we observed in the current chronic schizophrenia cohort may be more easily detectable among individuals at earlier stages of the illness relative to healthy individuals. If this notion is substantiated by future research, *NRG–ErbB* pathway gene expression may serve, in part, as a useful peripheral biomarker for staging of the illness and possibly assist in the identification of those at greatest risk for TRS.

## Ethics Statement

All the participants provided written informed consent and the study protocol was approved by the Melbourne Health Human Research Ethics Committee (MHREC ID 2012.069). The study complied with the Declaration of Helsinki and its subsequent revisions.

## Author Contributions

MSM, CB, IE, GC, and SS designed the study and wrote the protocol. MM, TL, and GC conducted the lab experiments. MM managed the literature searches and analyses and wrote the first draft of the manuscript. All authors contributed to and have approved the final manuscript.

## Conflict of Interest Statement

The authors declare that the research was conducted in the absence of any commercial or financial relationships that could be construed as a potential conflict of interest.
