# Supplementary material for: Peripheral Transcription of NRG-ErbB Pathway Genes Are Upregulated in Treatment-Resistant Schizophrenia
Source: Front Psychiatry. 2017 Nov 6;8:225. doi: 10.3389/fpsyt.2017.00225 (PMC5681734; doi:10.3389/fpsyt.2017.00225)
Supplement: Supplementary file 1 [file data_sheet_1.docx]

**SUPPLEMENTARY MATERIAL**

**Title: Peripheral Transcription of *NRG-ErbB* Pathway Genes is Upregulated in Treatment-Resistant Schizophrenia**

| **Table of contents** | **Page no.** |
| --- | --- |
| **Supplementary Methods** | **2-4** |
|  |  |

**Supplementary Figures**

| **Figure S1** | LDH toxicity assay results at 24 hours and 7 days post-clozapine exposure | **5** |
| --- | --- | --- |
| **Figure S2** | Expression of reference genes between treatment-resistant schizophrenia patients vs healthy controls | **6** |
| **Figure S3** | Expression of reference genes after 24hours clozapine exposure | **7** |
| **Figure S4** | Expression of reference genes after 7days clozapine exposure | **8** |
| **Figure S5** | Quantile-quantile plots of log10 transformed NRQ values for the genes | **9** |
| **Figure S6** | Expression of detectable genes after 24hours clozapine exposure | **10** |
| **Figure S7** | Expression of detectable genes after 7 days clozapine exposure | **11** |

**Supplementary Tables**

| **Table S1.** | TaqMan probes and primer sequence for quantification and normalization of different NRG-ErbB pathway gene expression | **12** |
| --- | --- | --- |
| **Table S2.** | Spearman’s correlation between different NRG-ErbB pathway gene expressions with clozapine plasma level, chlorpromazine equivalent antipsychotic exposure, age of onset | **13** |
| **Table S3.** | Spearman’s correlation between NRG-ErbB pathway genes with PANSS positive, negative, disorganized, excited, depression score and total score | **14** |
| **Table S4.** | Normalized relative quantities (NRQ) of detectable NRG-ErbB pathway genes by remission status | **15** |
| **References** |  | **16** |

**SUPPLEMENTARY METHODS**

***Blood sampling method***

Blood was collected after overnight fasting and processed according to the Australian Imaging, Biomarker and Lifestyle (AIBL) blood collection and processing protocol. A trained nurse or scientist undertook the blood draw at existing facilities within the local hospital and pathology services. The blood sample was then transported to the AIBL laboratory at the Mental Health Research Institute for further processing. Briefly, blood samples were collected in EDTA tubes (for DNA), PAXgene® Blood RNA tubes for total RNA extraction. Blood processing was done within 2 hours of venipuncture under sterile conditions. The gel serum tubes were allowed to clot before processing (at least 20 minutes following venipuncture). After the tubes were filled with blood, they were inverted 8-10 times for mixing. They were kept at ambient temperature until processed. The PAXgene tubes were allowed to stand upright at room temperature for 24 hours in the dark and then overnight at -20°C. The EDTA tubes were centrifuged for 15 minutes at 1500 x g at 20°C. All the tubes were stored at -80°C until further processing.

***Cell culture method***

The clozapine used in this experiment was obtained from Sigma-Aldrich (St. Louis, Missouri, USA). The concentration of clozapine used in this experiment was determined from the mean plasma concentration of clozapine found in the cohort of treatment-resistant schizophrenia patients (1.2µM or 384ng/mL). Clozapine induces PBMC death at higher concentrations (5x10^-6^ to 2.5x10^-5^ M) dosage (1). In spite of this LDH toxicity assay was performed at baseline, 24 hours and 7 day time periods.

PBMCs were incubated in RPMI-1640 medium (Sigma-Aldrich; St. Louis, Missouri, USA) supplemented with L-glutamine (0.3g/L) and sodium bicarbonate (2g/L), penicillin (100units/mL), streptomycin (100µg/mL), 10% fetal bovine serum and 1.2µM of clozapine at 37°C in 5% CO_2_. They were seeded at a concentration of 2 million cells per well (1x10^6^ cells/mL) in triplicate in six-well plates and treated with 1.2µM of clozapine for 24 hours and 7 days. Absolute ethanol was used to prepare 10mM clozapine solution and it was diluted with media to prepare 0.48mM clozapine solution. From this 5µL was added to each well so that each well is exposed with 1.2µM of clozapine. Each control well was exposed with media only. Cells were exposed to clozapine for 24 hours and 7 days. Total RNA was extracted at both time points.

***RNA extraction from cultured PBMCs and quality control***

Total RNA was extracted from 24 hours and 7 day time periods from both clozapine exposed and control cells using PureLink™ RNA Mini Kit (Life Technologies®, ThermoFisher Scientific™, Waltham, MA, USA) using a standard protocol. Briefly, cells were lysed with using lysis buffer containing 1% 2-mercaptoethanol. After the pellets were dispersed and cells appear lysed they were homogenized by passing the lysate 5-10 times through an 18-gauzge syringe needle. Next, RNA purification was performed using wash buffer I and II and RNA was eluted in 30µL of RNase-free water. All the RNA tubes were stored at -80°C until further use. The quality of extracted RNA was checked for RIN and concentration using the Agilent® RNA ScreenTape assay with the Agilent 2200 TapeStation system (Agilent Technologies, Santa Clara, CA, USA).

***Reverse Transcription of mRNA***

After extraction, RNA was reverse transcribed to cDNA. Then, cDNA was used as a template for RT-qPCR using master-mix and gene specific validated Taqman assays from Applied Biosystems, Foster City, California, USA. For cDNA synthesis, total RNA (200 ng) was denatured for 5 min at 65°C, and then reverse transcribed using the SuperScript® IV First-Strand cDNA Synthesis Kit (Invitrogen, Carlsbad, California, USA) in a 20μL reaction volume containing 1× Reverse Transcription SSIV buffer, 10mM dNTP mixture, 50µM random hexamers, 100mM DTT, 40 U/µl RNase OUT™ Recombinant RNase Inhibitor and 200 U/µl SuperScript® IV Reverse Transcriptase. The reaction (40 cycles) will be incubated at 23°C for 10 min, followed by 55°C for 10 min, 80°C for 10 min. To remove RNA, 1 µl of *E.coli* RNase H was added to each reaction mixture and incubated at 37°C for 20min and finally held at 4°C. All cDNA samples were stored at -20°C until qPCR analysis. In each plate, one no template control and one RT negative were used for quality control purposes.

***Real-Time Quantitative Polymerase Chain Reaction (RT-qPCR)***

Gene expression was performed using FAM-MGB TaqMan® gene expression probes (Invitrogen, Foster city, CA, USA) in 192.24 Dynamic Arrays IFC in Fluidigm® BioMark™ HD system (South San Francisco, CA, USA) at the MHTP Medical Genomics Facility (Monash Health Translation Precinct, Hudson Institute of Medical Research, Clayton, VIC, Australia). All the samples were run in duplicates in two independent 192.24 BioMark IFC arrays to ensure there was no technical variability. Prior to gene expression, quality control was performed. All the samples qualified except one and that was excluded from the further experiment. The RT negatives showed a very high Ct value indication absence or low genomic DNA contamination and the ‘no template’ control did not show any amplification.

The FAM-MGB, TaqMan gene expression assays are provided as 20x forward and reverse primer and probe mixes. Each primer is at a concentration of 18µM and the probe is at a concentration of 4µM. The TaqMan assays were selected from the Single Cell Genomics Taqman Library at the Single Cell Genomics Centre (MHTP Medical Genomics Facility, Monash Health Translation Precinct, Clayton, VIC, Australia). Inventoried assays (TaqMan®, Invitrogen, Foster city, CA, USA) were used for all genes of interest and reference (housekeeping) genes. Supplementary table (2) contains the list of the probes and primers.

Pre-amplification was done to increase the number of copies of each gene to detectable levels as detailed in Gene Expression Preamp with Fluidigm® Preamp Master Mix and TaqMan® Assays Quick Reference PN 100-5876B1. To reduce bias, the pre-amplification procedure takes probes and primers of all genes of interest and makes a probe-primer pool and then all the samples get the equal amount of the mixture for amplification. Taqman assays were firstly pooled by combining 4μL of each of the 24 20X TaqMan assays and 304μL C1 DNA suspension buffer for a final volume of 400μL. The final concentration of each assay was 0.2X (180nM).

Pre-amplification allows multiplex amplification. 3.75μL of Sample Pre-Mix (Life Technologies TaqMan® PreAmp Master Mix and Pooled Taqman assays) was combined with 1.25μL of each of the cDNA samples, RT-negative samples, and ‘no template’ water controls for a final reaction volume of 5μL per sample. An additional no template control (by the gene expression facility) was also included and all samples were pre-amplified for 14 cycles. Following pre-amplification, reaction products were diluted 1:5 by adding 20μL C1 DNA suspension buffer to the final 5μL reaction volume for a total volume of 25μL.

Assays and Samples were combined in a 192.24 Dynamic Array IFC according to Fluidigm® 192.24 Real-Time PCR Workflow Quick Reference PN 100-6170. Briefly, 3μL of each assay at a final concentration of 10X was added to each assay inlet port and 3μL of diluted sample to each sample inlet port according to the Chip Pipetting Map. For unused sample inlets, 2.2uL of sample premix and 1.8uL of water per inlet were used. The data were analyzed with Fluidigm Real-Time PCR analysis software (V4.1.1).

Normalized relative quantities of different gene isoforms (mRNA) were calculated relative to the geometric mean of two reference genes, beta actin (*ACTB*) and ubiquitin-c (*UBC*). The reference genes were selected based on a previously reported gene expression experiment conducted on post-mortem brain tissue in schizophrenia patients and controls (2). The relative quantities of *ACTB* and *UBC* were not significantly different between the groups in the clinical cohort. Two of the four reference genes (*ABL-1* and *SDHA*) were found to significantly differ in the clinical cohort and therefore was not used as reference genes for normalization. For the *in-vitro* cohort, *ABL-1* was stable at 24hours and *ACTB* was stable at 7days clozapine post exposure and so were used for normalization at specific time points.

**
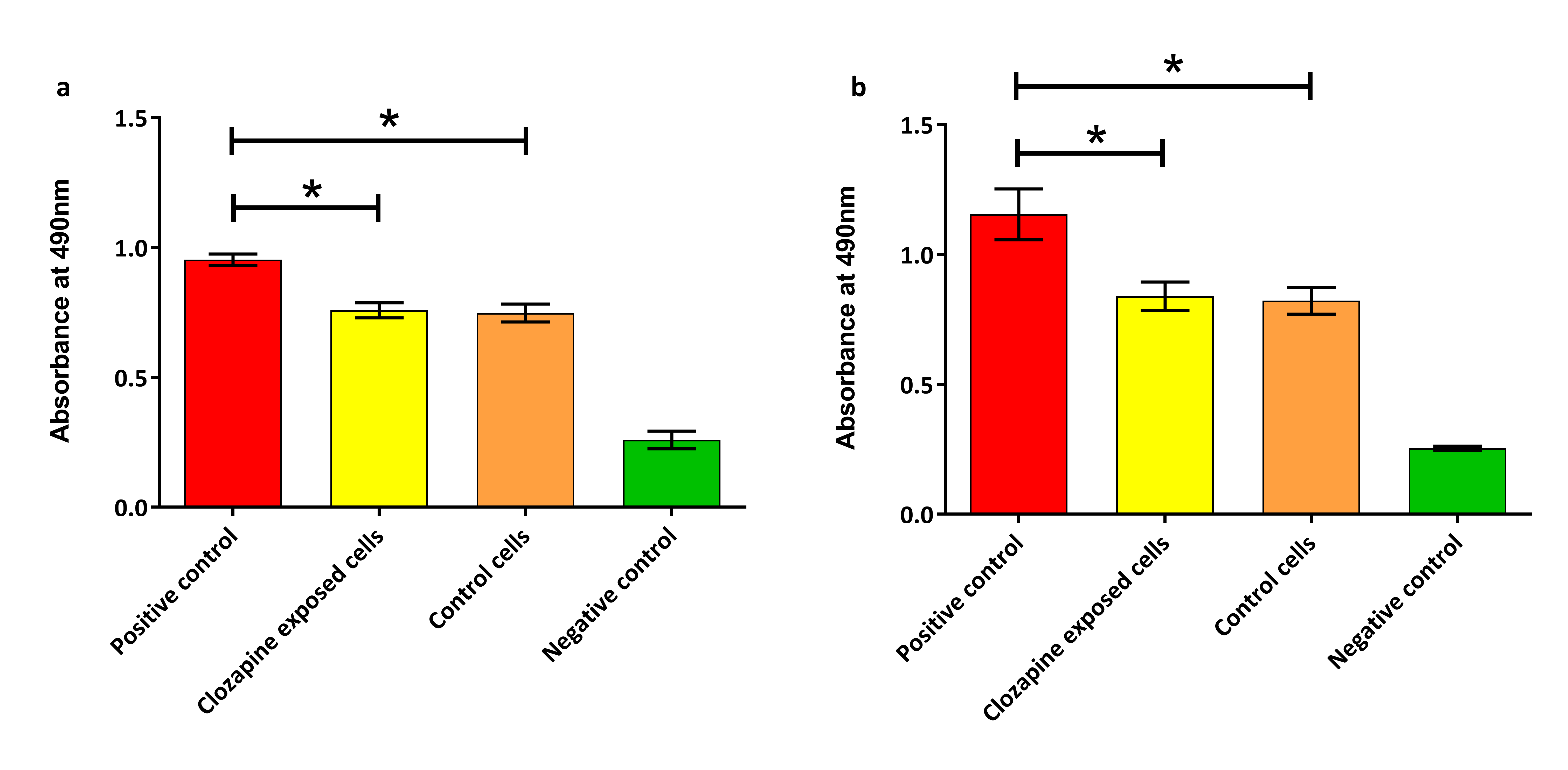
**

**Supplementary Figure S1:** LDH toxicity assay results in cultured PBMCs, (a) 24 hour post exposure and (b) 7-days post exposure. *P=0.001


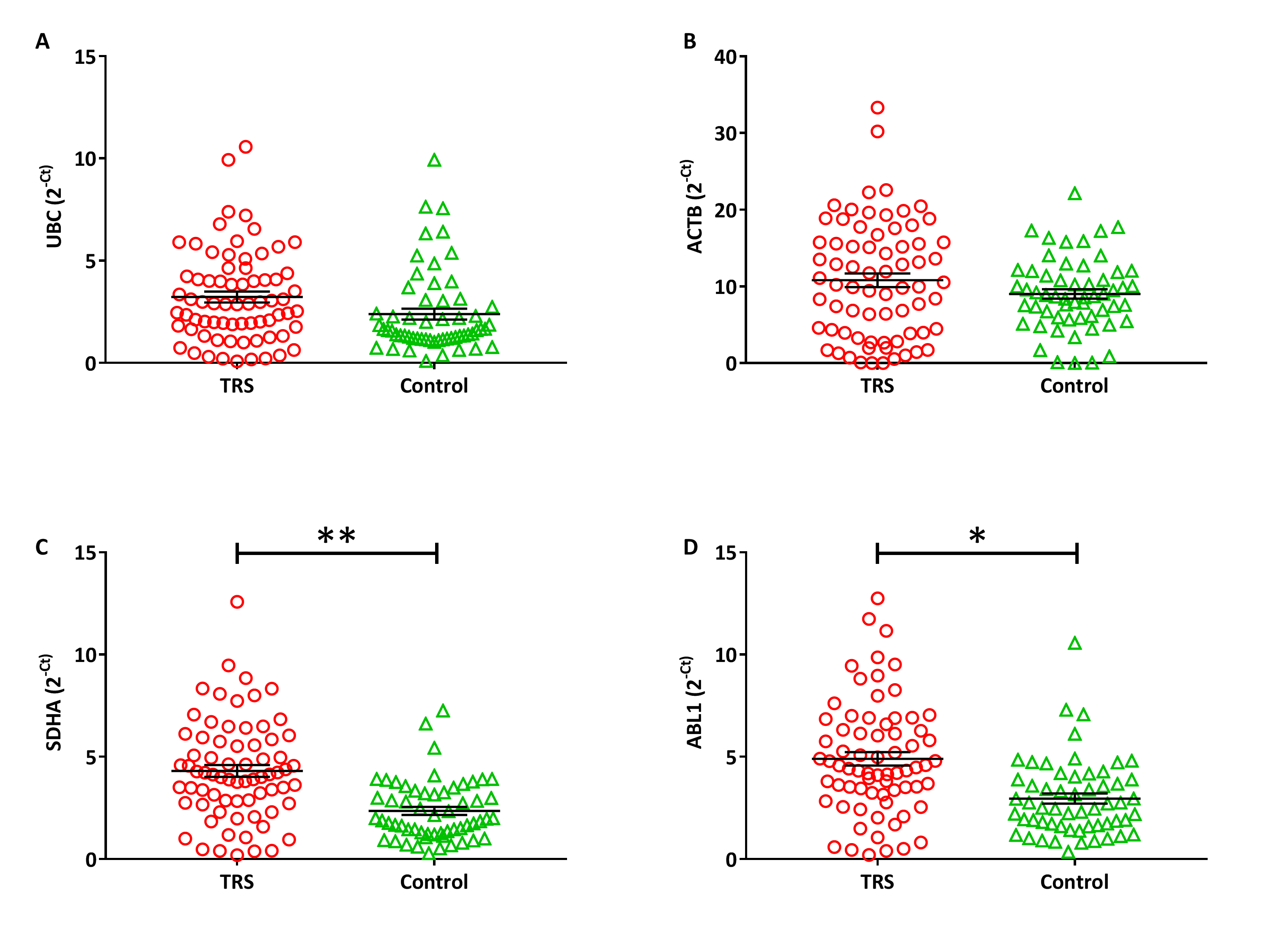


**Supplementary Figure S2**: Expression of reference genes in treatment-resistant schizophrenia (TRS) patients vs healthy controls; (**A**) *UBC* (t=-1.877, df =126, P=0.063), (**B**) *ACTB* (t=0.140, df=126, P=0.889), (**C**) *SDHA* (t=-4.20, df=87.383, P=0.000051), (**D**) *ABL-1* (t=-3.466, df=105.563, P=0.001). Error bars represent mean ± s.e.m. *P<0.05, **P<0.001.


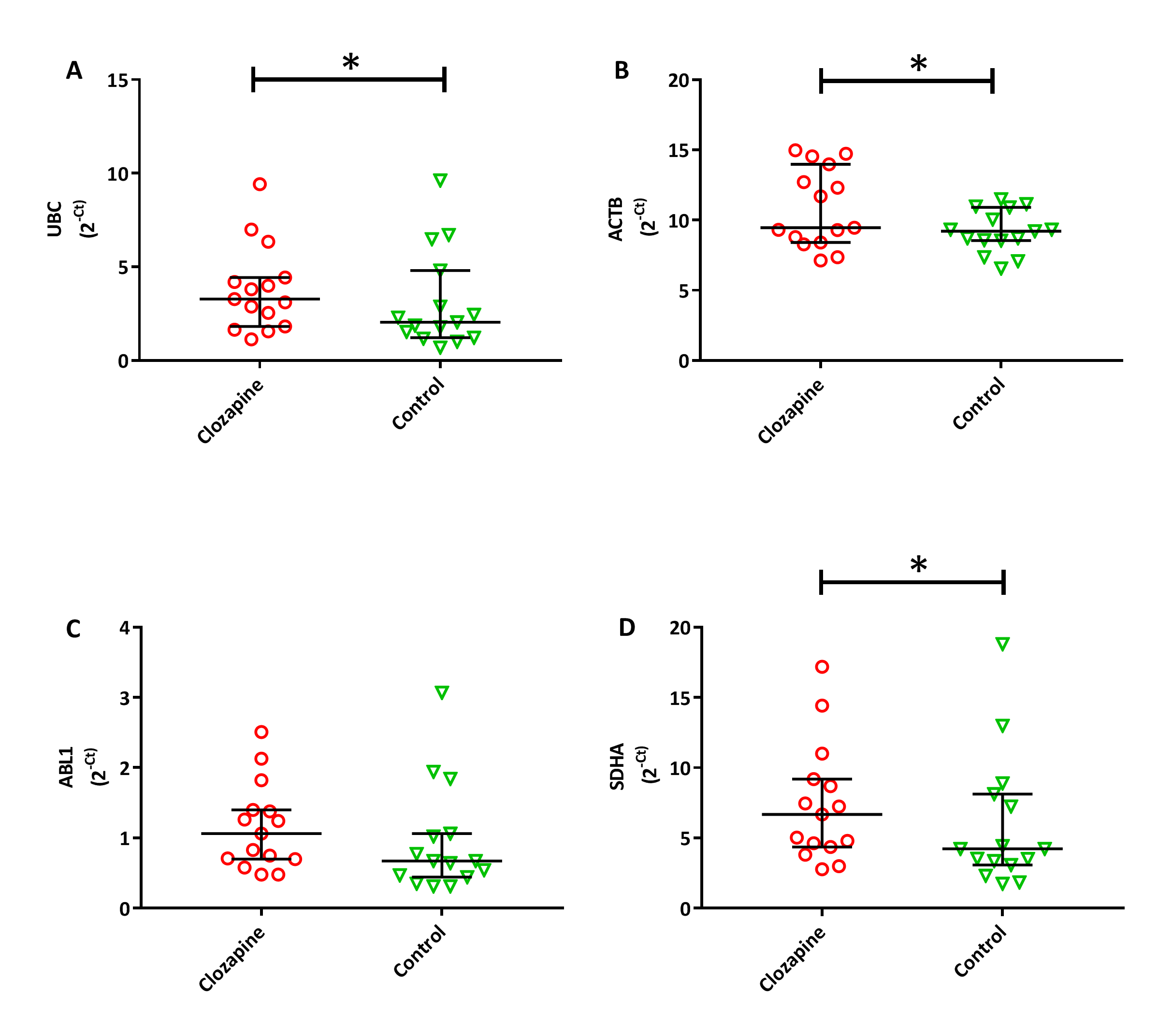


**Supplementary Figure S3:** Expression of reference genes after 24hours clozapine exposure. Wilcoxon signed rank test (matched pair, α=0.05, N=15) was used to measure the difference in reference gene expression between clozapine exposed and control cells; (**a**) *UBC* (W=-2.385, P=0.017), (**b**) *ACTB* (W=2.499, P=0.012), (**c**) *ABL-1* (W=-1.931, P=0.053), (**d**) *SDHA* (W=-2.158, P=0.031). Error bars represent median ± interquartile range. *P<0.05.


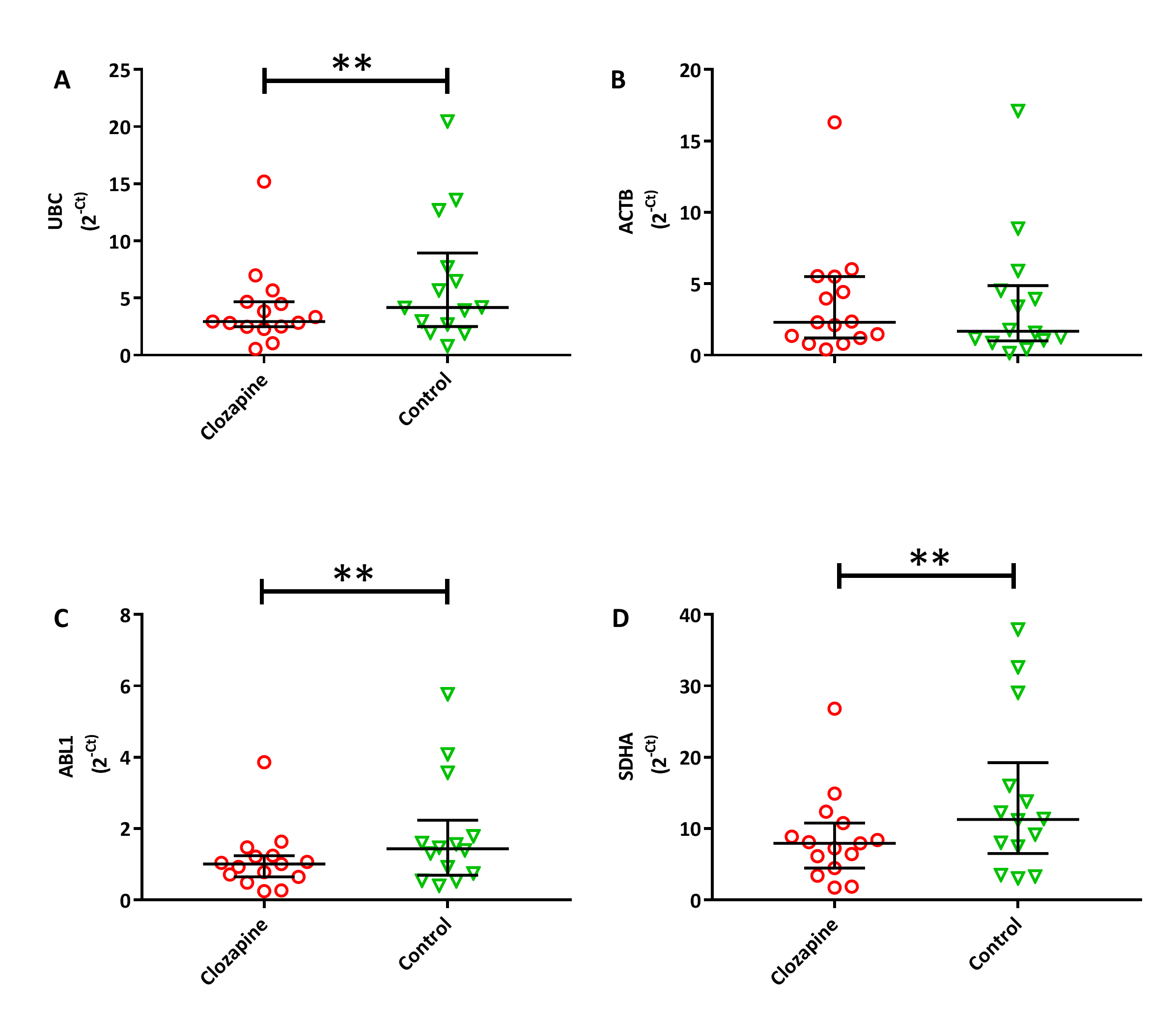


**Supplementary Figure S4:** Expression of reference genes after 7 days clozapine exposure. Wilcoxon signed rank test (matched pair, α=0.05, N=15) was used to measure the difference in reference gene expression between clozapine exposed and control cells; (**a**) *UBC* (W=3.045, P=0.002), (**b**) *ACTB* (W=-0.722, P=0.470), (**c**) *ABL-1* (W=3.233, P=0.001), (**d**) *SDHA* (W=3.233, P=0.001). Error bars represent median ± interquartile range. **P<0.01.

**
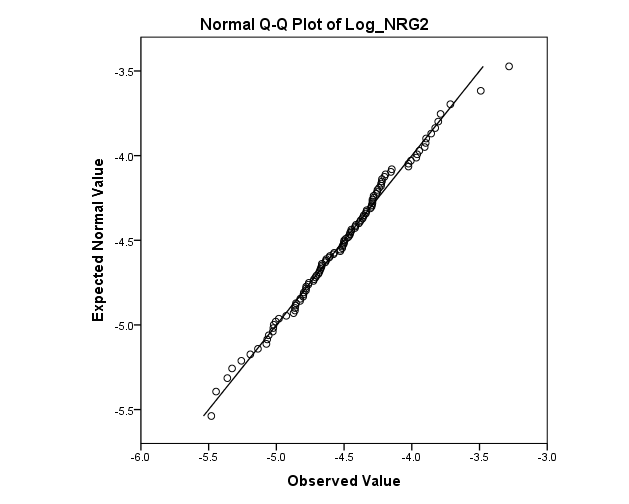
** **
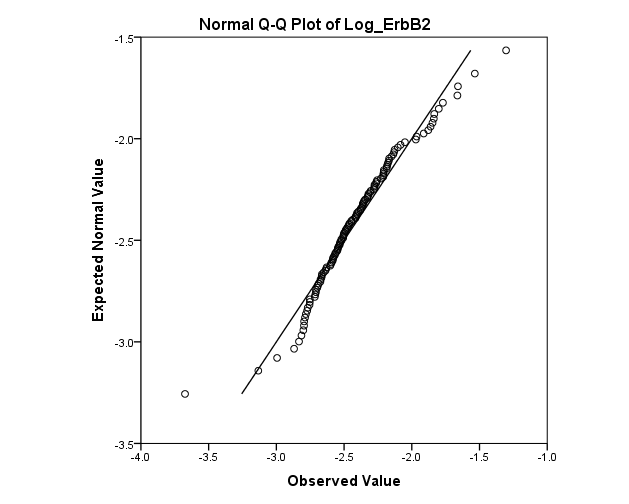
**
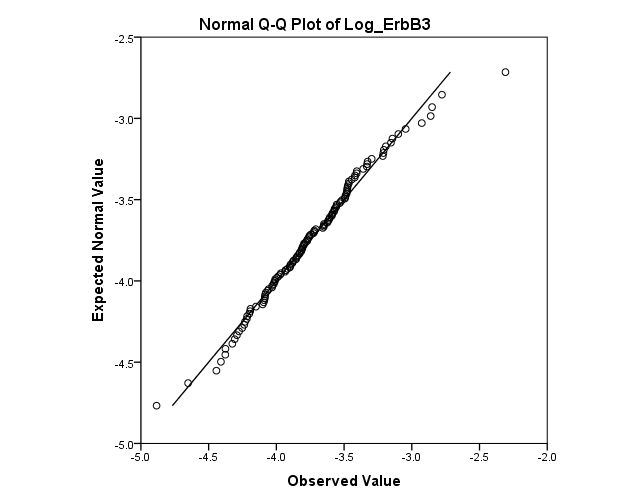


**C**

**B**

**A**

**E**

**F**

**D**

**
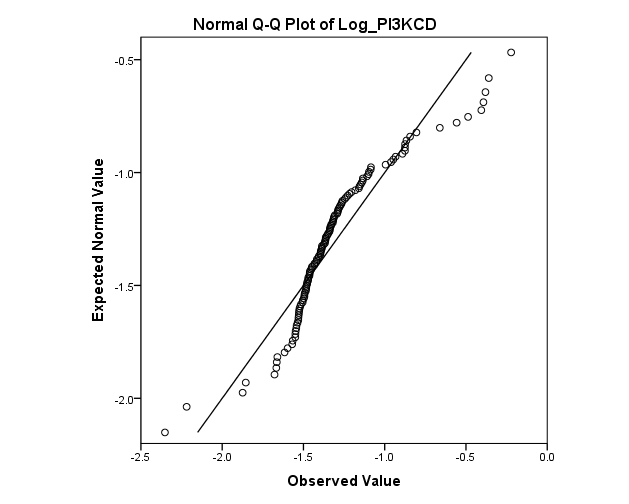
** **
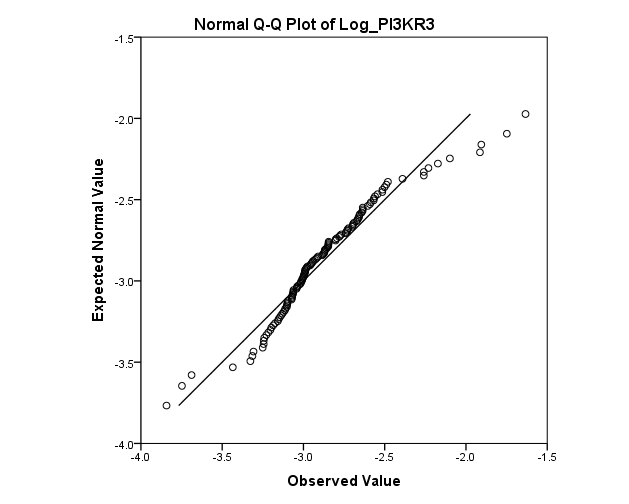
**
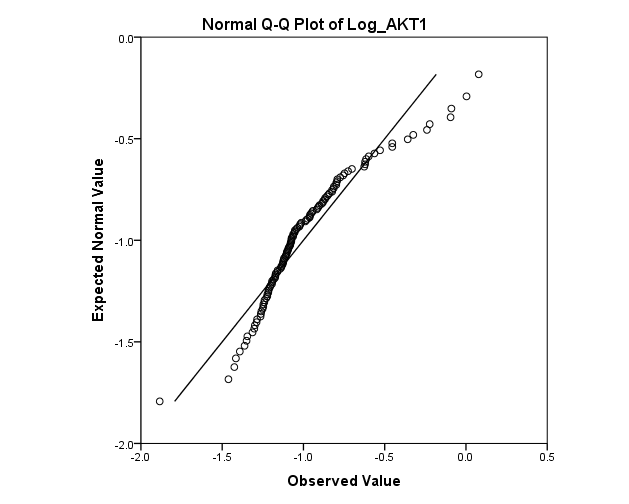


**I**

**H**

**G**

**
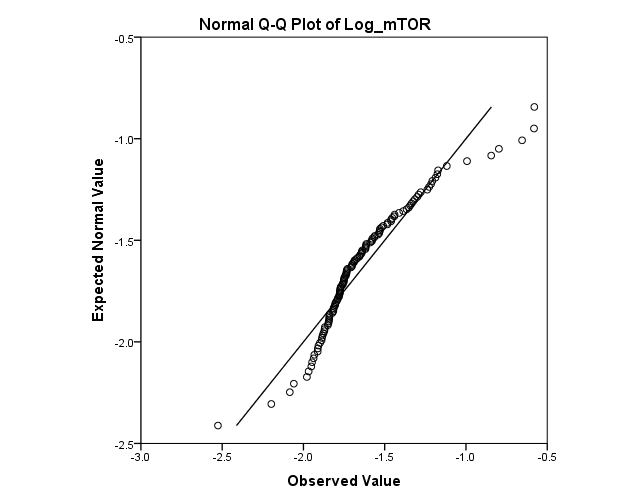
** **
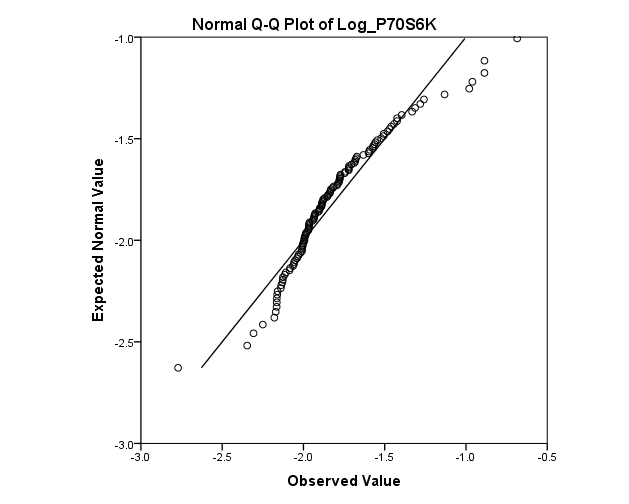
**
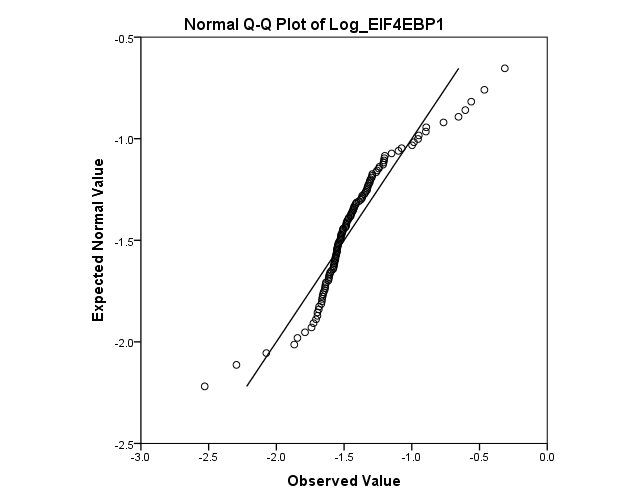


**Supplementary Figure S5**: Quantile-quantile plots of Log10 transformed NRQ values for (A) *NRG2* (SW=0.992, df=112, P=0.789), (B) *ErbB2* (SW=0.961, df=127, P=0.001), (C) *ErbB3* (SW=0.987, df=126, P=0.279), (D) *PIK3CD* (SW=0.887, df=128, P<0.001) and (E) *PIK3R3* (SW=0.941, df=127, P<0.001), (F) *AKT1* (SW=0.910, df=127, P<0.001), (G) *mTOR* (SW=0.908, df=127, P<0.001), (H) *P70S6K* (SW=0.925, df=127, P<0.001), (I) *eIF4EBP1* (SW=0.889, df=127, P<0.001). SW=Shapiro-Wilk test.

**
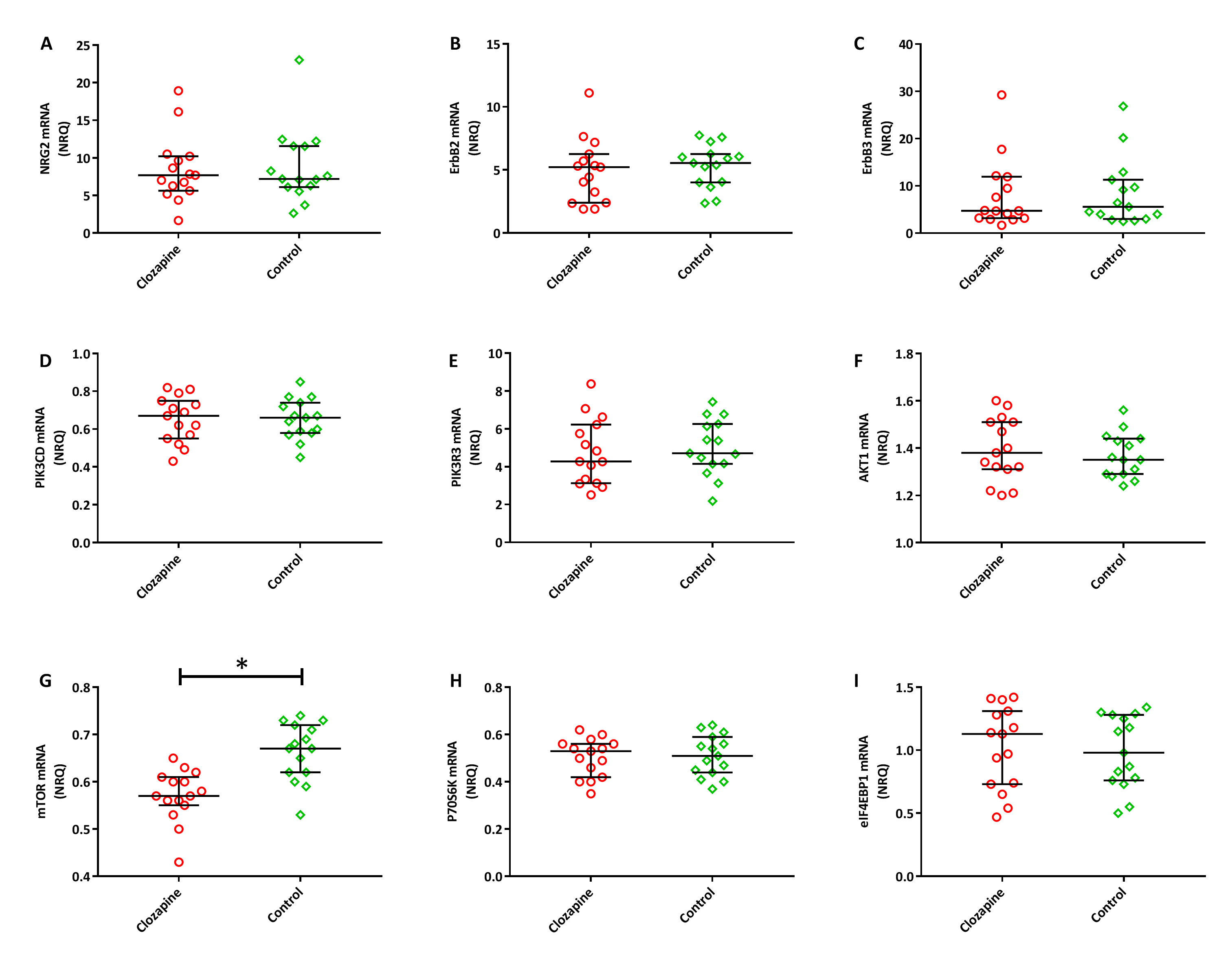
**

**Supplementary Figure S6:** Expression of detectable gene transcripts after 24hours clozapine exposure. Wilcoxon signed rank test (matched pair) was used to measure the difference in the normalized quantity of transcripts between clozapine exposed and control cells; (**A**) *NRG2* (W=0.341, P=0.733), (**B**) *ErbB2* (W=1.874, P=0.061), (**C**) *ErbB3* (W=0.966, P=0.334), (**D**) *PIK3CD* (W= 0.001, P=1.0), (**E**) *PIK3R3* (W= 1.193, P=0.233), (**F**) *AKT1* (W=-1.079, P=0.281), (**G**) *mTOR* (W=3.408, P=0.001), (**H**) *P70S6K* (W=1.59, P=0.112), (**I**) *eIF4EBP1* (W=-0.341, P=0.733). Error bars represent median ± interquartile range. *P<0.01.

**
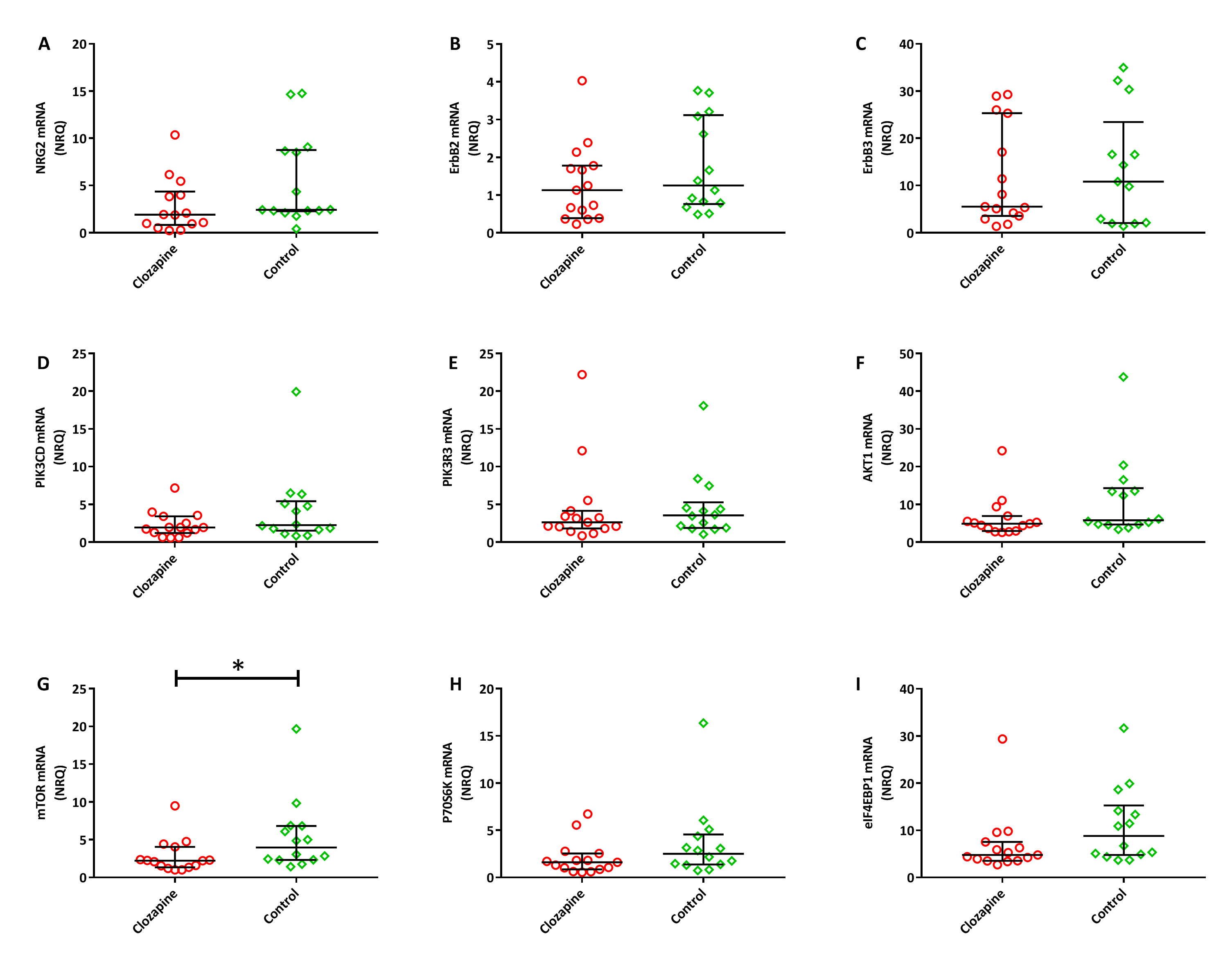
**

**Supplementary Figure S7:** Expression of detectable gene transcripts after 7 days clozapine exposure. Wilcoxon signed rank test (matched pair) was used to measure the difference in the normalized quantity of transcripts between clozapine exposed and control cells; (**A**) *NRG2* (W=1.922, P=0.055), (**B**) *ErbB2* (W=1.601, P=0.109), (**C**) *ErbB3* (W=1.915, P=0.056), (**D**) *PIK3CD* (W=1.664, P=0.096), (**E**) *PIK3R3* (W=1.161, P= 0.245), (**F**) *AKT1* (W=1.726, P=0.084), (**G**) *mTOR* (W=2.80, P=0.005), (**H**) *P70S6K* (W=1.664, P=0.096), (**I**) *eIF4EBP1* (W=1.726, P=0.084). Error bars represent median ± interquartile range. *P<0.01.

| **No.** | **Gene** | **Isoforms detected** | **Inventoried assay** |
| --- | --- | --- | --- |
| 1 | *NRG2* | DON1, HRG2, NTAK | Hs00171706_m1 |
| 2 | *ErbB1/EGFR* | HER1, NISBD2, PIG61,mENA | Hs01076090_m1 |
| 3 | *ErbB2* | CD340, HER-2, HER-2/neu, HER2, MLN 19, NEU, NGL, TKR1 | HS01001580_m1 |
| 4 | *ErbB3* | HER3, LCCS2, MDA-BF-1, c-erbB-3, c-*ErbB3*, *ErbB3*-S, p180-*ErbB3*, p45-s*ErbB3*, p85-s*ErbB3* | Hs00176538_m1 |
| 5 | *ErbB4* | ALS19, HER4, p180*ErbB4* | Hs00955525_m1 |
| 6 | *PIK3CD* | APDS, IMD14, P110DELTA, PI3K, p110D | Hs00908666_m1 |
| 7 | *PIK3R3* | p55, p55-GAMMA, p55PIK | Hs01103591_m1 |
| 8 | *AKT1* | AKT, CWS6, PKB, PKB-ALPHA, PRKBA, RAC, RAC-ALPHA | Hs00178289_m1 |
| 9 | *mTOR* | FRAP, FRAP1, FRAP2, RAFT1, RAPT1, SKS | Hs00234508_m1 |
| 10 | *P70S6K* | PS6K, S6K, S6K-beta-1, S6K1, STK14A, p70 S6KA, p70(S6K)-alpha, p70-S6K, p70-alpha | Hs00356367_m1 |
| 11 | *eIF4EBP1* | 4E-BP1, 4EBP1, BP-1, PHAS-I | Hs00607050_m1 |
| 12 | *ABL-1* | ABL proto-oncogene 1 | Hs01104728_m1 |
| 13 | *SDHA* | Succinate Dehydrogenase Complex Flavoprotein Subunit A | Hs00188166_m1 |
| 14 | *ACTB* | beta-actin | Hs99999903_m1 |
| 15 | *UBC* | ubiquitin C | Hs00824723_m1 |

**Supplementary Table S1:** Inventoried TaqMan assays (Applied Biosystems, Foster City, CA, USA) for quantification and normalization of NRG-ErbB pathway genes of interest expression. The geometric mean of *UBC* and *ACTB* were used for transcript normalization.

| **Transcripts** | **Age of Onset, years** | **Clozapine Plasma level (µg/L)** | **Chlorpromazine equivalent antipsychotic exposure (excluding clozapine)** |
| --- | --- | --- | --- |
| *NRG2* | 0.130  (0.315) | 0.117  (0.360) | 0.010  (0.936) |
| *ErbB2* | 0.019  (0.879) | -0.014  (0.911) | 0.083  (0.493) |
| *ErbB3* | -0.051  (0.679) | 0.094  (0.444) | -0.020  (0.869) |
| *PIK3CD* | 0.033  (0.791) | -0.016  (0.893) | 0.082  (0.500) |
| *PIK3R3* | -0.063  (0.607) | -0.024  (0.847) | 0.057  (0.639) |
| *AKT1* | 0.003  (0.983) | 0.023  (0.853) | 0.076  (0.532) |
| *mTOR* | 0.003  (0.981) | 0.033  (0.785) | 0.012  (0.922) |
| *P70S6K* | -0.004  (0.977) | 0.018  (0.880) | 0.017  (0.891) |
| *eIF4EBP1* | -0.198  (0.104) | 0.022  (0.855) | 0.071  (0.558) |

**Supplementary Table S2:** Spearman’s correlation (raw P-value) between different gene transcripts level with age of onset, clozapine plasma level and chlorpromazine equivalent antipsychotic exposure (excluding clozapine).

| **Transcripts** | **Positive score** | **Negative score** | **Disorganized score** | **Excitement score** | **Depression score** | **Total score** |
| --- | --- | --- | --- | --- | --- | --- |
| *NRG2* | -0.138 (0.667) | 0.010 (0.964) | 0.117  (0.667) | 0.077  (0.784) | -0.125 (0.667) | -0.086 (0.778) |
| *ErbB2* | -0.069 (0.784) | 0.161 (0.667) | 0.051  (0.828) | 0.289  (**0.014**, 0.667) | -0.073 (0.784) | 0.097 (0.687) |
| *ErbB3* | -0.066 (0.787) | 0.212 (0.667) | 0.044  (0.834) | 0.196  (0.667) | -0.013 (0.964) | 0.132 (0.667) |
| *PIK3CD* | 0.108 (0.667) | 0.070 (0.784) | 0.146  (0.667) | 0.061  (0.787) | 0.042 (0.834) | 0.124 (0.667) |
| *PIK3R3* | 0.006 (0.964) | 0.103 (0.667) | 0.037  (0.852) | 0.230  (0.667) | -0.141 (0.667) | 0.061 (0.787) |
| *AKT1* | 0.117 (0.667) | 0.119 (0.667) | 0.183  (0.667) | 0.142  (0.667) | 0.005 (0.964) | 0.160 (0.667) |
| *mTOR* | 0.045 (0.834) | 0.105 (0.667) | 0.166  (0.667) | 0.103  (0.667) | -0.107 (0.667) | 0.121 (0.667) |
| *P70S6K* | 0.093 (0.895) | -0.026 (0.667) | 0.121  (0.667) | 0.180  (0.667) | 0.142 (0.667) | -0.124 (0.701) |
| *eIF4EBP1* | 0.032 (0.874) | 0.176 (0.667) | 0.222  (0.667) | 0.183  (0.667) | -0.051 (0.828) | 0.178 (0.667) |

**Supplementary Table S3:** Spearman’s correlation (raw P-value, Benjamini-Hochberg adjusted P- value) between gene transcripts level with PANSS positive, negative, disorganized, excited, depression score and total score.

| **Gene transcripts** | **Remission**  **N; median (IQR)** | **Non-remission**  **N; median (IQR)** | **Raw P value** | **BH P value*** |
| --- | --- | --- | --- | --- |
| *NRG2* | 28; 3.82 (1.52-5.88) | 35; 3.01 (1.41-4.40) | 0.261 | - |
| *ErbB2* | 31; 3.85 (2.55-6.78) | 39; 3.68 (2.20-6.01) | 0.727 | - |
| *ErbB3* | 31; 2.58 (1.45-3.79) | 39; 2.10 (0.93-3.24) | 0.158 | - |
| *PIK3CD* | 31; 4.02 (3.45-7.34) | 40; 4.82 (3.43-7.62) | 0.557 | - |
| *PIK3R3* | 31; 1.34 (0.77-2.04) | 40; 1.33 (0.97-2.20) | 0.581 | - |
| *AKT1* | 31; 0.86 (0.73-1.61) | 40; 1.02 (0.79-1.72) | 0.523 | - |
| *mTOR* | 31; 1.99 (1.66-4.31) | 39; 2.29 (1.70-3.60) | 0.837 | - |
| *P70S6K* | 31; 1.47 (1.25-2.68) | 39; 1.67 (1.10-2.72) | 0.982 | - |
| *eIF4EBP1* | 31; 3.70 (2.77-5.69) | 39; 3.99 (2.92-5.61) | 0.502 | - |

**Supplementary Table S4:** Normalized relative quantities (NRQ) of detectable gene transcripts by positive symptom remission status. Positive symptom remission was defined as a score of ≤3 on four PANSS items (delusions, hallucinations, grandiosity and unusual thought content) (3). *Benjamini-Hochberg adjusted P-value

**References**

1. Chen ML, Tsai TC, Lin YY, Tsai YM, Wang LK, Lee MC, et al. Antipsychotic drugs suppress the AKT/NF-kappaB pathway and regulate the differentiation of T-cell subsets. *Immunol Lett* (2011) 140(1-2):81-91. doi: 10.1016/j.imlet.2011.06.011. PubMed PMID: 21763349.

2. Weickert CS, Sheedy D, Rothmond DA, Dedova I, Fung S, Garrick T, et al. Selection of reference gene expression in a schizophrenia brain cohort. *Aust N Z J Psychiatry* (2010) 44(1):59-70. doi: 10.3109/00048670903393662. PubMed PMID: 20073568; PubMed Central PMCID: PMCPMC2950262.

3. Wallwork RS, Fortgang R, Hashimoto R, Weinberger DR, Dickinson D. Searching for a consensus five-factor model of the Positive and Negative Syndrome Scale for schizophrenia. *Schizophr Res* (2012) 137(1-3):246-50. doi: 10.1016/j.schres.2012.01.031. PubMed PMID: 22356801; PubMed Central PMCID: PMC3351536.
